# Diabetes Mellitus and Related Admission Factors Among Hospitalized Patients in King Abdul-Aziz University Hospital in Jeddah, Saudi Arabia

**DOI:** 10.7759/cureus.25312

**Published:** 2022-05-25

**Authors:** Yasamen A Shikdar, Hala H Mosli, Nasrin A Shikdar, Rajaa M Alshanketi, Noorah A Shikdar, Raghdaa M Malebary, Wedyan M Aboznadah, Mohammad A Shikdar

**Affiliations:** 1 Internal Medicine/Diabetes and Endocrinology, Fakeeh College for Medical Sciences, Jeddah, SAU; 2 Internal Medicine/Diabetes and Endocrinology, King Abdul-Aziz University, Jeddah, SAU; 3 Internal Medicine, Althagher Hospital, Jeddah, SAU; 4 Internal Medicine, King Abdul-Aziz University, Jeddah, SAU

**Keywords:** complication, jeddah saudi arabia, hospitalized, diabetes mellitis, prevalence

## Abstract

Background

Diabetes mellitus (DM) is a rapidly increasing serious health problem that affects the population all over the world. The increasing prevalence of DM in Saudi Arabia is reflected in our hospital admissions as well. This study aimed to assess the proportion of DM (including type 1 and type 2 diabetes) among hospitalized patients and the reasons for admissions to the medical unit at King Abdul-Aziz University Hospital (KAUH) in Jeddah, Saudi Arabia.

Methods

We conducted a hospital record-based cross-sectional study at KAUH from January to April 2021. The study included all adult patients admitted to the internal medicine wards and isolation unit but excluded patients in the coronary care unit and those with gestational diabetes. We reviewed the medical records to collect demographic data, causes of admission, laboratory results, and outcomes.

Results

Among the hospitalized patients, 49.9% had DM. The most common associated risk factors and causes of admission among patients with DM were hypertension (HTN; 73.2%) and dyslipidemia (43.1%). Other less common reasons for admission were heart failure (20.6%), coronavirus disease-2019 (COVID-19; 17.8%), chronic kidney disease (CKD; 14.5%), pneumonia (12.3%), and stroke (10%). Dyslipidemia, HTN, CKD, diabetic ketoacidosis, heart failure, and need for intensive care unit (ICU) admission were significantly higher in diabetic patients as compared to patients without diabetes. HTN, dyslipidemia, CKD, heart failure, stroke, acute abdomen, and malignancy were significantly higher in patients with type 2 diabetes. Among diabetic patients, those with non-Saudi nationality, low hemoglobin level, dyslipidemia, pneumonia, sepsis, and requiring ICU admission had a greater risk of death.

Conclusions

The high burden of DM on the secondary healthcare level in Saudi Arabia highlights the need for effective diabetes prevention and treatment strategies in primary care and hospital outpatient settings. Such measures would help reduce the hospitalization rate and ease the healthcare system’s burden.

## Introduction

Diabetes mellitus (DM) is a chronic multisystem disorder influenced by a complex interaction of genetic, socioeconomic, and behavioral factors that impair insulin secretion from the pancreas resulting in insulin resistance [1]. Diabetic patients usually manifest high blood glucose levels and can have various complications that affect their blood vessels and nerves [1]. Complications can be microvascular (e.g., neuropathy, retinopathy, and nephropathy) and macrovascular (e.g., stroke and myocardial infarction [MI]) [1,2]. DM also causes immune dysfunction because hyperglycemia is toxic to cellular immunity. Therefore, patients with uncontrolled DM have a greater risk for morbidity and mortality than patients without DM [2].

Several hospital admissions are related to DM and its complications, and as the global incidence of DM grows, the disease consumes a growing amount of the national healthcare expenditure [2]. The diabetes epidemic will continue to grow if primary prevention is not implemented, and it is expected to become one of the world's leading causes of disability and mortality if left untreated [3-5]. According to the International Diabetes Federation, the Middle East and North Africa (MENA) region has the highest DM prevalence [6]. This spike is due to rapid economic development, urbanization, and lifestyle changes.

Furthermore, according to the World Health Organization, the Kingdom of Saudi Arabia (KSA) has the second-highest prevalence of DM in the Middle East region and seventh in the world [4]. In a systematic review, KSA had an incidence of 32.8% of type 2 diabetes in 2015, which is expected to increase to 45.36% by 2030 [5]. Epidemiological studies were done in KSA [1] and Libya [3], and both reported that DM patients had higher admission rates, longer hospital stays, and higher morbidity and mortality than patients without DM. Data regarding the prevalence of DM among patients and their reasons for admission to the KSA are limited. This study aimed to identify the proportion of DM (including type 1 and type 2 diabetes) and the reasons for admission among hospitalized patients in the medical unit at King Abdul-Aziz University Hospital (KAUH) in Jeddah, KSA.

## Materials and methods

We conducted a hospital record-based cross-sectional study to identify the proportion of DM and causes of admission among hospitalized patients in KAUH in Jeddah, KSA, from January to April 2021. The study was ethically approved by the Institutional Review Board (IRB) of KAUH. We reviewed the medical records of all adult patients admitted to internal medicine wards and the isolation units. Patients admitted to the coronary care unit and those with gestational diabetes were excluded from the study as they were not admitted under internal medicine wards. We collected demographic information, such as age, gender, nationality (i.e., Saudi or Non-Saudi), and any known medical issues by history such as diabetes, hypertension (HTN), chronic kidney disease (CKD), and dyslipidemia. We also recorded the reason for the current admission and laboratory results, such as glycated hemoglobin (HbA1c), hemoglobin, creatinine, microalbumin/creatinine ratio, total cholesterol, triglyceride, low-density lipoprotein (LDL), high-density lipoprotein (HDL), and thyroid-stimulating hormone (TSH). We recorded patient outcomes in terms of length of stay, intensive care unit (ICU) admission, surgery, and discharge or in-hospital death.

Statistical analysis

Data were analyzed statistically using IBM SPSS Statistics for Windows, Version 26.0 (IBM Corp., Armonk, NY). To assess the relationship between variables, qualitative data were expressed as numbers and percentages, and the chi-squared test (χ^2^) was used. Quantitative data were expressed as mean ± standard deviation, and non-parametric variables were tested using the Mann-Whitney test. Multivariate logistic regression analysis was done to assess the independent predictors (risk factors) of death among diabetic patients, and the odds ratio (OR) was determined at a confidence interval of 95%. A p-value of 0.05 was considered statistically significant.

## Results

The study population consisted of 800 patients (56.9% were women, and 43.1% were men) with a mean age of 55.6 ± 18.1 years. Most of the patients (54.8%) were Saudis. A total of 399 patients had DM (49.9%), and of those patients, 338 (84.7%) patients had type 2 diabetes and 164 of these DM patients (41.1%) were on insulin therapy. Of all studied patients, 693 (86.6%) patients did not require ICU admissions, while 102 (12.8%) patients were admitted to the ICU. Among those with diabetes, only 126 patients (31.6%) had controlled HbA1c, and the mean length of hospital stay for all patients was 10.8 ± 21.8 days, and 66 patients (8.3%) died in the hospital. Table 1 presents the demographic and clinical characteristics of the study population.

**Table 1:** Distribution of studied participants according to their demographic and clinical characters (n = 800) DM: Diabetes mellitus; GLP-1: Glucagon-like peptide 1; TG: Triglycerides; LDL: Low-density lipoprotein; HDL: High-density lipoprotein; TSH: Thyroid-stimulating hormone.

Variables	No. (%)
Age (years)	55.69 ± 18.11
Gender	
Female	345 (43.1)
Male	455 (56.9)
Nationality	
Non-Saudi	362 (45.3)
Saudi	438 (54.8)
Having DM	
No	401 (50.1)
Yes	399 (49.9)
DM type (No.: 399)	
Type 1	61 (15.3)
Type 2	338 (84.7)
DM treatment (No.: 399)	
Combined oral + GLP-1 agonist injection	2 (0.5)
Diet + insulin	1 (0.2)
Diet + oral hypoglycemic drugs	2 (0.5)
Insulin	164 (41.1)
Not on treatment	29 (7.3)
Oral hypoglycemic drugs	125 (31.3)
Oral hypoglycemic drugs + insulin	76 (19)
HbA1C (No.:399)	
Controlled	126 (31.6)
Not controlled	188 (47.1)
NA	85 (21.3)
Hospital course (No.: 800)	
Did not require ICU	693 (86.6)
Required ICU	102 (12.8)
Required surgery	5 (0.6)
Outcomes	
Deceased	66 (8.3)
Discharged	734 (91.8)
Length of hospital stay (days)	21.81 ± 10.8
Mean and SD of lab results	Normal ranges
HbA1C < 5.7 (%)	7.3 ± 2.17
Hemoglobin: 12.6-17.4 (g/dl)	11.26 ± 2.73
Creatinine: 0.67-1.17 (mg/dl)	271.94 ± 828.38
Total CHOL: 0-199 (mg/dl)	4.02 ± 1.27
TG: 0.00-150 (mg/dl)	1.46 ± 0.9
LDL: 0.00-100 (mg/dl)	2.99 ± 6.27
HDL ≥ 55 (mg/dl)	1.58 ± 7.48
TSH: 0.27-4.20 (uIU/ml)	5.17 ± 15.45

The most common associated risk factors and causes of admission among patients with DM were HTN (73.2%) and dyslipidemia (43.1%). The other causes of admission were heart failure (20.6%), coronavirus disease 2019 (COVID-19; 17.8%), CKD (14.5%), pneumonia (12.3%), and stroke (10%). End-stage renal disease (ESRD) was found in 9.5% of admissions, and other conditions found were urinary tract infection (UTI; 7%), renal failure (6.8%), sepsis (4%), diabetic ketoacidosis (DKA; 2.5%), malignancy (2%), transient ischemic attack (TIA; 1.5%), bleeding (1%), MI (0.8%), acute abdomen (0.8%), and meningoencephalitis (0.5%).

A comparison of comorbidities in patients with DM and those without DM is seen in Table 2. Not surprisingly, DM patients had a significantly increased risk of having dyslipidemia, HTN, CKD, DKA, heart failure, and ICU admissions than the patients without DM (p ≤ 0.05). Patients without DM had more incidence of acute abdomen and malignancy (p ≤ 0.05).

**Table 2:** Difference between diabetic and non-diabetic patients according to risk factors and causes of admission (n = 800) HTN: Hypertension; CKD: Chronic kidney disease; DKA: Diabetic ketoacidosis; TIA: Transient ischemic attack; ESRD: End-stage renal disease; UTI: Urinary tract infection; TG: Triglycerides; LDL: Low-density lipoprotein; HDL: High-density lipoprotein; TSH: Thyroid-stimulating hormone.

Variables	No DM (No.: 401)	DM (No.: 399)	χ^2^	p-value
	No. (%)	No. (%)		
HTN	131 (31)	292 (69)	131.8	<0.001
Dyslipidemia	51 (22.9)	172 (77.1)	91.87	<0.001
CKD	36 (38.3)	58 (61.7)	7.78	0.02
DKA	0 (0.0)	10 (100)	10.17	0.001
Acute coronary syndrome (MI)	4 (57.1)	3 (42.9)	0.13	0.709
Heart failure	30 (26.8)	82 (73.2)	28.37	<0.001
Stroke	29 (42)	40 (58)	1.98	0.159
TIA	5 (45.5)	6 (54.5)	0.09	0.755
Pneumonia	33 (40.2)	49 (59.8)	3.56	0.059
Renal failure	26 (49.1)	27 (50.9)	0.02	0.872
ESRD on dialysis	34 (47.2)	38 (52.8)	0.26	0.606
UTI	26 (48.1)	28 (51.9)	0.09	0.764
COVID-19	59 (45.4)	71 (54.6)	1.39	0.238
Acute abdomen	21 (87.5)	3 (12.5)	13.82	<0.001
Sepsis	10 (38.5)	16 (61.5)	1.46	0.227
Meningoencephalitis	6 (75)	2 (25)	2	0.157
Malignancy	27 (77.1)	8 (22.9)	10.68	0.001
Bleeding	8 (66.7)	4 (33.3)	1.33	0.248
Hospital course				
Did not require ICU	359 (51.8)	334 (48.2)	6.74	0.034
Required ICU	39 (38.2)	63 (61.8)		
Required surgery	3 (60)	2 (40)		
Length of hospital stay	9.89 ± 16.35	11.71 ± 26.16	0.71	0.474
Lab results	Normal ranges			
Hemoglobin: 12.6-17.4 (g/dl)	11.37 ± 2.84	11.15 ± 2.62	1.44	0.148
Creatinine: 0.67-1.17 (mg/dl)	194.18 ± 594.06	351.23 ± 1007.72	6.83	<0.001
Total CHOL: 0-199 (mg/dl)	4.11 ± 1.43	3.96 ± 1.34	1.04	0.295
TG: 0.00-150 (mg/dl)	1.44 ± 1.01	1.47 ± 0.82	0.71	0.476
LDL: 0.00-100 (mg/dl)	3.72 ± 10.78	2.63 ± 1.2	0.63	0.52
HDL ≥ 55 (mg/dl)	1.1 ± 0.37	1.81 ± 9.1	0.94	0.345
TSH: 0.27-4.20 (uIU/ml)	6.82 ± 19.2	3.96 ± 11.88	0.64	0.519
Outcomes				
Deceased	28 (42.4)	38 (57.6)	1.7	0.191
Discharged	373 (50.8)	361 (49.2)		

A comparison of comorbidities in type 1 vs. type 2 diabetes patients is depicted in Table 3. HTN, dyslipidemia, CKD, heart failure, stroke, acute abdomen, and malignancy were significantly higher in patients with type 2 diabetes (p ≤ 0.05) than in the patients with type 1 diabetes. Patients with type 1 diabetes had more DKA (p ≤ 0.05), which was expected.

**Table 3:** Difference between diabetes types according to risk factors and causes of admission (n = 399) HTN: Hypertension; CKD: Chronic kidney disease; DKA: Diabetic ketoacidosis; TIA: Transient ischemic attack; ESRD: End-stage renal disease; UTI: Urinary tract infection.

Variables	DM type 1 (No.: 61)	DM type 2 (No.: 338)	χ2	p-value
	No. (%)	No. (%)		
HTN	30 (7.1)	262 (61.9)	148.39	<0.001
Dyslipidemia	27 (12.1)	145 (65)	91.91	<0.001
CKD	7 (7.4)	51 (54.3)	9.7	0.045
DKA	10 (100)	0 (0.0)	122.68	<0.001
Acute coronary syndrome (MI)	1 (14.3)	2 (28.6)	0.79	0.673
Heart failure	10 (8.9)	72 (64.3)	29.41	<0.001
Stroke	2 (2.9)	38 (55.1)	6.13	0.046
TIA	2 (18.2)	4 (36.4)	1.77	0.413
Pneumonia	4 (4.9)	45 (54.9)	6.13	0.047
Renal failure	3 (5.7)	24 (45.3)	0.42	0.809
ESRD on dialysis	5 (6.9)	33 (45.8)	0.42	0.81
UTI	2 (3.7)	26 (48.1)	1.69	0.43
COVID-19	11 (8.5)	60 (46.2)	1.39	0.497
Acute abdomen	1 (4.2)	2 (8.3)	14.02	0.001
Sepsis	2 (7.7)	14 (53.8)	1.58	0.453
Meningoencephalitis	1 (12.5)	1 (12.5)	2.94	0.23
Malignancy	0 (0.0)	8 (22.9)	11.37	0.003
Bleeding	0 (0.0)	4 (33.3)	1.82	0.402
Hospital course				
Did not require ICU	49 (7.1)	285 (41.1)	7.98	0.092
Required ICU	12 (11.8)	51 (50)		
Required surgery	0 (0.0)	2 (40)		
Length of hospital stay	8.66 ± 13.28	12.27 ± 27.84	1	0.317
Outcomes				
Deceased	6 (9.1)	32 (48.5)	1.71	0.424
Discharged	55 (7.5)	306 (41.7)		

Table 4 presents outcomes in patients with DM. Patients of older age, of non-Saudi nationality, those with lower hemoglobin levels, higher creatinine levels, and longer hospital stays had significantly higher mortality (p ≤ 0.05). Dyslipidemia, pneumonia, sepsis, or ICU requirement were also associated with a significantly higher incidence of death (p ≤ 0.05). Outcomes among patients with diabetes were not significantly affected by gender, treatment types, HbA1c, total cholesterol, triglycerides, LDL, HDL, TSH levels, and all causes of admission except dyslipidemia, pneumonia, or sepsis (p ≥ 0.05).

**Table 4:** Relationship between the outcomes among diabetic patients and their demographic and clinical characteristics (n = 399) DM: Diabetes mellitus; GLP-1: Glucagon-like peptide 1; TG: Triglycerides; LDL: Low-density lipoprotein; HDL: High-density lipoprotein; TSH: Thyroid-stimulating hormone; HTN: Hypertension; CKD: Chronic kidney disease; DKA: Diabetic ketoacidosis; TIA: Transient ischemic attack; ESRD: End-stage renal disease; UTI: Urinary tract infection.

Variables	Outcomes			
	Deceased (No.: 38) No. (%)	Discharged (No.: 361) No. (%)		
Age (years)	66.79 ± 15.17	61.31 ± 15.27	2.38	0.017
Gender				
Female	11 (7.1)	144 (29.9)	1.73	0.188
Male	27 (11.1)	217 (88.9)		
Nationality				
Non-Saudi	26 (13.5)	167 (86.5)	6.76	0.009
Saudi	12 (5.8)	194 (94.2)		
DM type (No.: 399)				
Type 1 (No.: 61)	6 (9.8)	55 (90.2)	0.008	0.928
Type 2 (No.: 338)	32 (9.5)	306 (90.5)		
DM treatment (No.: 399)				
Combined oral + GLP-1 agonist injection (No.: 2)	1 (50)	1 (50)	6.15	0.407
Diet + insulin (No.: 1)	0 (0.0)	1 (100)		
Diet + oral hypoglycemic drugs (No.: 2)	0 (0.0)	2 (100)		
Insulin (No.: 164)	18 (11)	146 (89)		
Not on treatment (No.: 29)	4 (13.8)	25 (86.2)		
Oral hypoglycemic drugs (No.: 125)	9 (7.2)	116 (92.8)		
Oral hypoglycemic drugs + insulin (No.: 76)	6 (7.9)	70 (92.1)		
Mean and SD of lab results	Normal ranges			
HbA1C < 5.7 (%)	7.21 ± 2.11	8.09 ± 2.27	1.67	0.093
Hemoglobin: 12.6-17.4 (g/dl)	9.72 ± 2.68	11.3 ± 2.57	3.55	<0.001
Creatinine: 0.67-1.17 (mg/dl)	549.67 ± 1065.72	330.4 ± 100089	2.17	0.03
Total CHOL: 0-199 (mg/dl)	3.42 ± 1.57	4.01 ± 1.31	1.45	0.146
TG: 0.00-150 (mg/dl)	1.59 ± 0.83	1.46 ± 0.83	0.72	0.467
LDL: 0.00-100 (mg/dl)	2.73 ± 1.03	2.62 ± 1.22	0.46	0.64
HDL ≥ 55 (mg/dl)	1.06 ± 0.28	1.85 ± 9.36	0.08	0.931
TSH: 0.27-4.20 (uIU/ml)	3.87 ± 5.69	3.97 ± 12.42	0.85	0.393
Length of hospital stay (days)	33.18 ± 57.62	9.46 ± 19.02	4.33	<0.001
Risk factors and causes of admission				
HTN (No.: 292)	26 (8.9)	266 (91.1)	0.48	0.486
Dyslipidemia (No.: 172)	24 (14)	148 (86)	6.88	0.009
CKD (No.: 58)	5 (8.6)	53 (91.4)	0.11	0.943
DKA (No.: 10)	0 (0.0)	10 (100)	1.08	0.299
Acute coronary syndrome (MI) (No.: 3)	0 (0.0)	3 (100)	0.31	0.573
Heart failure (No.: 82)	9 (11)	73 (89)	0.25	0.615
Stroke (No.: 40)	3 (7.5)	37 (92.5)	0.21	0.646
TIA (No.: 6)	0 (0.0)	6 (100)	0.64	0.432
Pneumonia (No.: 49)	11 (22.4)	38 (77.6)	10.83	0.001
Renal failure (No.: 27)	2 (7.4)	25 (92.6)	0.15	0.698
ESRD on dialysis (No.: 38)	3 (7.9)	35 (92.1)	0.12	0.719
UTI (No.: 28)	1 (3.6)	27 (96.4)	1.23	0.266
COVID-19 (No.: 71)	7 (9.9)	64 (90.1)	0.01	0.915
Acute abdomen (No.: 3)	0 (0.0)	3 (100)	0.31	0.573
Sepsis (No.: 16)	4 (25)	12 (75)	4.63	0.031
Meningoencephalitis (No.: 2)	0 (0.0)	2 (100)	0.21	0.646
Malignancy (No.: 8)	1 (12.5)	7 (87.5)	0.08	0.772
Bleeding (No.: 4)	0 (0.0)	4 (100)	0.42	0.514
Hospital course				
Did not require ICU (No.: 334)	16 (4.8)	318 (95.2)	56.05	<0.001
Required ICU (No.: 63)	22 (34.9)	41 (65.1)		
Required surgery (No.: 2)	0 (0.0)	2 (100)		

Table 5 shows that having a non-Saudi nationality, low hemoglobin levels, dyslipidemia, pneumonia, sepsis, and requiring ICU admission were independent predictors (i.e., risk factors) of death among diabetic patients (confidence interval: 95%; p ≤ 0.05).

**Table 5:** Multivariate logistic regression analysis of the independent predictors (risk factors) of death among diabetic patients (n = 399) N.B.: Wald = Wald test ("Wald" column) is used to determine the statistical significance of each of the independent variables. B = The regression slope or unstandardized coefficient and is the amount by which we predict that SciSore changes for an increase of one unit in wealth.

Variables	B	Wald	P-value	OR (CI: 95%)
Age	0.02	2.22	0.135	0.97 (0.94-1)
Nationality	0.97	4.49	0.034	0.37 (0.15-0.92)
Hemoglobin level	0.28	8.78	0.003	1.33 (1.1-1.6)
Creatinine level	0.001	0.03	0.86	0.99 (1-0.101)
Length of hospital stay	0.01	3.66	0.055	0.98 (0.97-1)
Dyslipidemia	1.02	5.17	0.023	2.79 (1.15-6.79)
Pneumonia	1.6	9.01	0.003	4.97 (1.74-14.19)
Sepsis	0.92	1.58	0.208	2.51 (0.59-10.54)
Hospital course	1.63	12.61	<0.001	5.13 (2.08-12.67)

## Discussion

DM is a rapidly increasing serious health problem that affects the global population. The prevalence of DM in KSA has increased 10-fold in the last 30 years [3]. This increase is reflected in our hospital admissions. Our study is one of the most comprehensive to date that describes the characteristics of hospitalized patients with and without DM. Overall, dyslipidemia, HTN, CKD, DKA, heart failure, and ICU admission were significantly higher in patients with diabetes as compared to patients without diabetes. In addition to diabetes, these factors increased the risk of hospitalization and longer hospital stays as supported by multiple previous reports [2,7,8].

In the present study, nearly half of the total admissions to the medical unit were patients with DM. This high proportion conveys the impact of diabetes on our community. A similar study in 2000 by Akbar et al. in our hospital reviewed 1006 admitted patients to the medical unit and showed only 17% prevalence of diabetic patients [1]. This remarkable increase in numbers is an alarming sign of a major health crisis, which necessitates prompt additional action to promote comprehensive control programs to prevent the further increase in the diabetic population, harm, and financial burden to the country. Another study from Kuwait Al-Sabah Hospital in 2010 by Al-Adsani et al. showed a 40.6% prevalence of diabetic patients hospitalized. This result is similar to our results [9].

Most of our patients had type 2 diabetes (84.7%), which can be explained by the rapid economic development and lifestyle changes. While only 15.3% of patients admitted had type 1 diabetes, this is more than a three-fold increase from the previous study at the same institution that found only 2% of the patients admitted had type 1 diabetes [1]. In our study, most of the patients (41.3%) were on insulin therapy, which provides an insight into their average HbA1c levels. Furthermore, 31.4% of DM patients used oral antihyperglycemic agents (OAHs), and 19% required insulin in addition to OAHs. Recent guidelines recommend glucagon-like peptide 1 receptor agonists early in the disease course for high-risk patients, but only 0.5% of patients in our study with type 2 diabetes were using this therapy [10].

Unfortunately, 7.3% of DM patients were not receiving any medications related to diabetes, which indicates a poor level of community awareness about diabetes, its complications, and the importance of being on a proper treatment regimen. Only a few DM patients (0.7%) were trying to control their blood glucose using diet alone; a similar proportion was reported by a previous study [11]. In our study, only 31.6% of patients with diabetes had good control with HbA1c < 7. In 2019, a study on outpatient diabetes treatment in our hospital also showed high numbers of poorly controlled diabetes in this population (68.31%) [12].

Patients with diabetes and HTN represented 73.2% of our study population. A study from China on hospitalized older patients with DM also reported a high prevalence of HTN (64.4%) [13]. Another study in older adults with DM also showed a high prevalence of HTN (81.9%) [14]. The prevalence of dyslipidemia among diabetic patients was 43.1%, which was lower than the incidence of dyslipidemia reported in a previous study from the northern region of KSA (66%) [15]. That same study showed that 39.0% of patients with DM had specifically high LDL cholesterol (LDL-C) levels [15]. Lipid profile levels in patients with DM and those without were not significantly different. A study in China showed that 61.1% of diabetes patients had LDL levels < 100 mg/dL [16]. Another report from Nepal demonstrated almost similar results, with 56.3% of DM patients having target LDL levels [17].

Of our diabetic patients, 9.5% had known ESRD on regular dialysis, and 6.8% had new renal failure. A study in the United States on DM patients showed that CKD prevalence was 43.5% [18]. In our study, 52.8% of patients with ESRD were on dialysis. An earlier study in the United Arab Emirates found that 44.0% of patients had moderately increased albuminuria [19]. Another US study showed the prevalence of elevated urine albumin excretion (i.e., >30 mg/g) to be 32.2% [18]. In KSA, a study on dialysis patients at one center in Tabuk found that diabetic nephropathy was the most common cause of ESRD, accounting for 30.4% of cases [20]. The prevalence of nephropathy associated with diabetes is very high in the MENA region [21].

According to our study, the most common reason for admission and risk factors in type 2 DM was dyslipidemia, followed by heart failure. DM is known as a major risk factor for cardiovascular events. These issues are caused by impaired glucose metabolism, and the resultant hypoglycemia or hyperglycemia increases the risk of arrhythmias, sudden death, or other cardiovascular events [22-24]. A study demonstrated that the risk of sudden cardiac death was at least two-fold higher in diabetic patients compared to those without diabetes [25]. Another study found that renal failure was the most common cause of admission among diabetic patients [26]. COVID-19 pneumonia was another reason for admission among patients with DM in our study, given that COVID-19 is often more serious in people with different comorbidities. Other studies reported that sepsis was the most common cause of admission among diabetic patients [27].

When we compared hospitalization in DM and non-DM patients, DM patients had longer hospital stays. This result aligns with a previous study [26]. Metabolic derangements, DM severity, and associated complications are predictors for longer hospital stays and mortality [26]. We found no significant association between death and DM, which contrasts with a previous study during the COVID-19 pandemic that found a higher mortality rate among patients with DM [28]. On multivariate logistic regression analysis to assess the risk factors of death among diabetic patients, low hemoglobin levels, dyslipidemia, pneumonia, sepsis, and ICU admission were independent predictors of death among diabetic hospitalized patients. A previous study reported that a history of diabetes was associated with worse outcomes in cardiomyopathy patients and was a predictor of mortality [29].

Strengths and limitations

Our study is one of the more comprehensive studies to date to describe the characteristics of hospitalized patients with and without DM. The study highlights the most common risk factors affecting public health. However, our study was limited by its cross-sectional design, which may reveal associations between variables but not causal relationships.

## Conclusions

Nearly half of the patients hospitalized during the study period had DM. Dyslipidemia, HTN, CKD, diabetic ketoacidosis, heart failure, and need for ICU admission were significantly higher in diabetic patients as compared to patients without diabetes. HTN, dyslipidemia, CKD, heart failure, stroke, acute abdomen, and malignancy were significantly higher in patients with type 2 diabetes. Risk factors for death were non-Saudi nationality, low hemoglobin levels, dyslipidemia, pneumonia, sepsis, and ICU admission. DM places a significant strain on secondary health care in the KSA. Effective primary care, preventative measures, and treatment strategies for diabetic patients may reduce these preventable hospitalizations.
